# Description of three new *Triaenodes* species from Fiji (Trichoptera, Leptoceridae)

**DOI:** 10.3897/zookeys.244.4034

**Published:** 2012-11-19

**Authors:** Kjell Arne Johanson, Maertha Eriksson, Hanna Müller, Helena Puranen Li

**Affiliations:** 1 Department of Entomology, Swedish Museum of Natural History, Box 50007, SE-104 05 Stockholm, Sweden

**Keywords:** Caddisflies, Terrestrial Arthropod Survey of Fiji, new species, taxonomy

## Abstract

The following three new species are illustrated and described from Fiji: *Triaenodes rebellus* Eriksson & Johanson, **sp. n.**, *Triaenodes oscitus* Müller & Johanson, **sp. n.**, and *Triaenodes forcipatus* Puranen Li & Johanson, **sp. n.** All species are endemic to Viti Levu. A key to the male *Triaenodes* species of Fiji is provided. With this report, the number of *Triaenodes* species known from Fiji is doubled.

## Introduction

In number of species, Trichoptera (caddisflies) constitute the second largest monophyletic group of aquatic animals (Malm et al. submitted). About 13.500 species are described so far, of which about 15% are classified in the family Leptoceridae Leach, 1815, which globally is ranked as the 2nd largest family in the order (Morse 2012). With 276 described species (Morse 2012) the leptocerids is the family in the Australian Region comprising the highest species diversity. The species of the family are characteristic in having adults with very long antennae, two rows of setae dorsally on the mesonotum, and narrow forewings. The sistergroup to the Leptoceridae is presently not identified, but based on phylogenetic analyses derived from molecular data the family is hypothesized related to Calamoceratidae, Atriplectidae, Odontoceridae and Philorheithridae (Malm et al. submitted). In the Australian Region, the Leptoceridae are easily distinguished from these families in the absence of preapical tibial spurs on the mid legs, from Philorheithridae by the long antennae, from Philorheithridae and Odontoceridae by having separate R1 and R2 at the forewing margin, and from Calamoceratidae and Philorheithridae by the narrower forewings (Neboiss 1986). The family comprises two subfamilies Leptocerinae Leach, 1815 and Triplectidinae Ulmer, 1906. Triaenodini is one of 11 tribes within Leptocerinae, and comprises the three genera *Adicella* McLachlan, 1877, *Erotesis* McLachlan, 1877 and *Triaenodes* McLachlan, 1865 (Malm and Johanson 2011). *Triaenodes* is known from all major faunal biogeographic regions and about 240 extant species have been described so far (Morse 2012, Johanson et al. 2011), and the extinct *Triaenodes fossilis* Wichard & Barnard, 2005 dated to Eocene, described from Baltic amber, represents the only fossil species in the genus. With 78 previously recorded species, the diversity is higher in the Australian Region compared to other regions. More than half of these (44 species) were described by Neboiss and Wells (1998) as endemic to Australia. So far two species are known from Vanuatu (Johanson et al. 2011), four from Solomon Islands (Kimmins 1957), 21 from New Guinea (Morse 2012), and three from Fiji. The first record of of the genus from Fiji was given by Mosely (1934) who described *Triaenodes dubius* Mosely, 1934 from Wainganitu. Banks (1936) described a second species, *Triaenodes manni* Banks, 1936, from Wainganitu. The third species *Triaenodes fijianus* Mosely, 1941 was described from Viti Levu. This paper adds three more species of the genus from Fiji.

## Material and methods

The study is based on five males collected in the Terrestrial Arthropod Survey of Fiji project (Evenhuis and Bickel 2005) funded by the US National Science Foundation and the Schlinger Foundation. In this project Trichoptera were collected in Malaise traps situated at 47 localities (Johanson and Oláh 2012) on the four major islands Viti Levu, Vanua Levu, Taveuni Island and Kadavu Island between September 21 2002 and January 5 2005. The *Triaenodes* specimens covered in this report were collected from four different localities at Viti Levu Island. The material is stored in 80% alcohol. Right wing pairs of the holotype of all new species and non-types of previously described species were removed, mounted on slides in glycerol and photographed using the Lumenera InfinityX digital camera mounted on an Olympus SZX12 stereomicroscope. The Extended Focus Option in the DeltaPix Insight software was used to create high-resolution photos with high focus depth. The abdomens were cleared in hot 8% KOH for about one hour. The abdomens were dehydrated in absolute alcohol and temporarily mounted in Euparal on a microscope slide before examination and drawing. All drawings were produced using a pencil on plain white A4 paper sheets using a drawing tube mounted on a Leitz Ortholux II. After the drawings were completed the abdomens were returned to the alcohol vial with the rest of the animal. Each pencil illustration was digitalized in a scanner at low resolution and thereafter used as a background layer in Adobe^©^ Photoshop^©^ 8.0. The illustrations were completed after being re-drawn on a new layer using a Wacom drawing pad before the background layer was deleted.

The nomenclature applied to the genitalic morphology follows that of Malm and Johanson (2011). Specimens in this study are deposited in the following repositories:

**FNIC** Fiji National Insect Collection, Suva, Fiji (currently held at BPBM)

**NHRS** Swedish Museum of Natural History, Stockholm, Sweden

The geographical setting of the localities are presented in Johanson and Oláh (2012).

## Descriptions

### Triaenodes rebellus

Eriksson & Johanson sp. n.

urn:lsid:zoobank.org:act:CC66BD00-165A-446E-BF77-EC1CAEEECAD9

http://species-id.net/wiki/Triaenodes_rebellus

Figs 1 , 4–8

#### Diagnosis.

*Triaenodes rebellus* resembles the New Guinean species *Triaenodes mondoanus* Kimmins, 1962 by having the dorsal right posterior margin of segment IX strongly produced posteriorly into a needle-shaped process. The new species is easily separated from *mondoanus* by the upper part of tergum X being about as long as the cerci, not much longer than cerci as in *Triaenodes mondoanus*; and the basomesal process and dorsal branch of each coxopodite is present, while absent in *mondoanus*.

#### Description, male.

**Figures 1–3.**
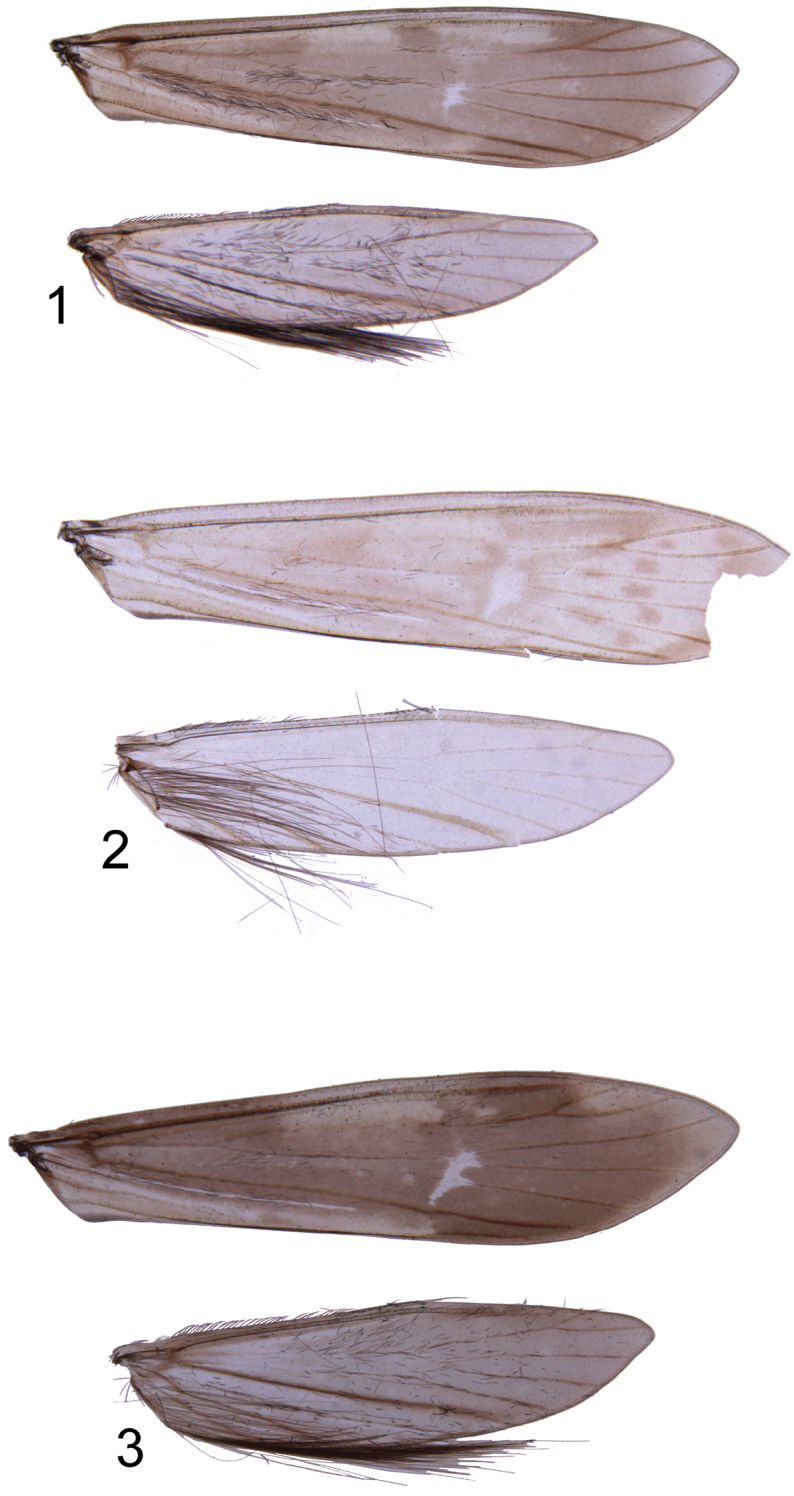
Right wings of holotypes. **1** *Triaenodes rebellus* sp. n. **2** *Triaenodes oscitus* sp. n. **3** *Triaenodes forcipatus* sp. n.

Wings (Fig 1). Forewing 5.2 mm, hind wing 4.3 mm (N=1). Forewing: stem of M absent; forks I and V present; wing membrane with pale area along apical margin, mid-anterior margin, and basal two-thirds of posterior margin. Hyaline area present at anastomosis. Hind wing: uniformly gray without apparent patterns.

**Figures 4–8.**
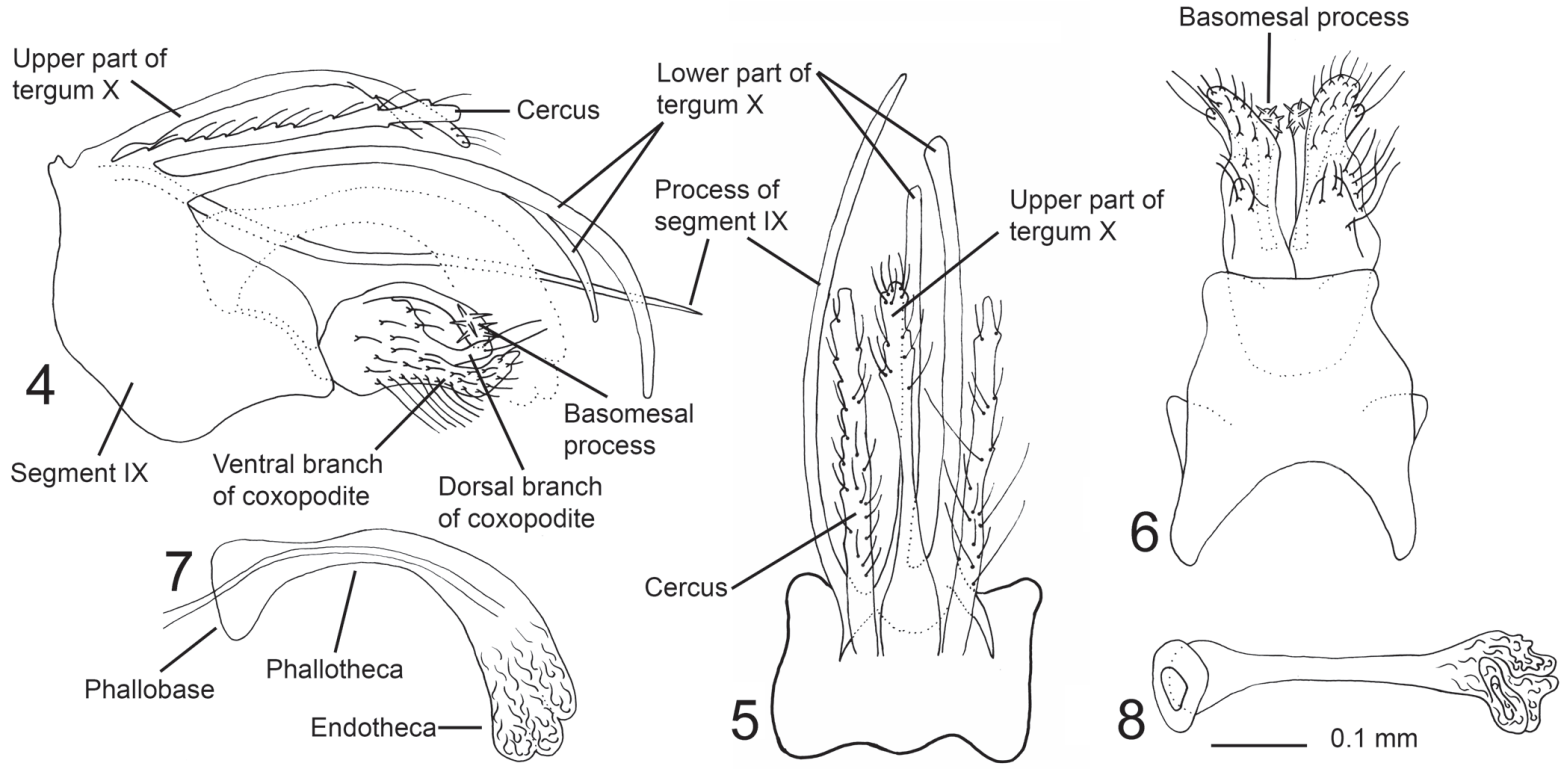
*Triaenodes rebellus* sp. n., holotype. **4** genitalia lateral view **5** genitalia dorsal view **6** genitalia ventral view **7** phallus lateral view **8** phallus ventral view.

Genitalia (Figs 4–8). Segment IX wide, asymmetric; in lateral view with ventral part produced posteriorly, anterior margin slightly convex, ventral margin concave; in dorsal view almost rectangular, anterior margin with central part slightly produced anterad; in ventral view broad anterior half and slightly narrower posterior half separated by narrow incision, anterior margin of segment IX deeply concave, lateral margins slightly concave, posterior margin straight. Dorsal right posterior margin of segment IX strongly produced posterad, forming almost straight, needle-shaped process exceeding processes of tergum X (visible in dorsal view and right lateral view), bow-shaped, gently curved mesally. Cerci straight, thin, elongate; covered with long, robust, and short, weak setae. Tergum X with well-developed upper and lower part, upper part about as long as cerci; forming central, slender, elongate, slightly dorsoventrally flattened process, setose at apical one-third; in lateral view with basal half almost straight, curving ventrally from mid-length (Fig. 4); in dorsal view slender, hourglass-shaped, with irregular lateral margins at distal one-third. Lower part of tergum X bilobed from base, each lobe thin, strongly elongate, significantly longer than cerci, right lobe slightly shorter than left lobe and tuboid at apex, left lobe slightly club-shaped at apex; in lateral view each lobe curved ventrally; in dorsal view almost parallel. Coxopodites in lateral view, with basal half almost circular, each divided at mid-length into dorsal and ventral branch, posterior margin above dorsal branch undulating. Each dorsal branch about one-third as long as total length of each coxopodite, originating from mid-height of basal part, tube-shaped, with two long apical setae; weakly bent dorsally at mid-length in lateral view; diverging apically in ventral view. Ventral branch of each coxopodite abundantly setose, more densely posteriorly; in lateral view bent dorsad at mid-length, apical part thin; in ventral view widest at base, lateral margin undulating, mesal margin sigmoid, apically diverging. Each basomesal process slender, apically club-shaped with abundant thick spines; in lateral view curved ventrally, reaching as far out as apex of dorsal branch; in ventral view straight or weakly curving mesally. Phallic organ about as long as dorsal part of tergum X; strongly curved ventrally immediately before mid-length, phallobase subtriangular, phallotheca slender immediately after phallobase, uniformly widening apically; endotheca apparently trilobed, membranous, without spines.

#### Material examined.

Holotype male: Fiji: Viti Levu Island, Naitasiri Province, Nakobalevu Mt., rainforest, 18°03'S, 178°25'E, 340 m, Malaise trap, 22.ix–9.x.2002, leg. M. Irwin, E. Schlinger & M. Tokoka’a. [FNIC, alcohol]

#### Etymology.

*Rebellus*, from Latin *rebellis*, insurgent in English, referring to genitalia being armed by many pointed processes.

### Triaenodes oscitus

Müller & Johanson sp. n.

urn:lsid:zoobank.org:act:72DEDCDE-DE32-4177-B248-28AA3F894EE0

http://species-id.net/wiki/Triaenodes_oscitus

Figs 2 , 9–13

#### Diagnosis.

The species resembles *Triaenodes rebellus* sp. n. in the shape of the ventral branch of the coxopodites and basomesal processes, but dorsal branch of each coxopodite is absent. The dorsal appendages of *oscitus* resemble those of *Triaenodes grifo* Malicky, 2005, but the upper part of tergum X of *oscitus* is longer.

#### Description, male.

Wings (Fig. 2). Forewing damaged, hind wing 4.1 mm (N=1).

Forewing: stem of M absent; forks I and V present; apex missing in holotype; membrane pale, except in distal one-third with two almost vertical series of dark spots, and a larger dark patch above vertical series. Hyaline area at anastomosis. Basal two-thirds with irregular pale fields. Hind wing: uniformly gray, with faint spots at apical half.

**Figures 9–13.**
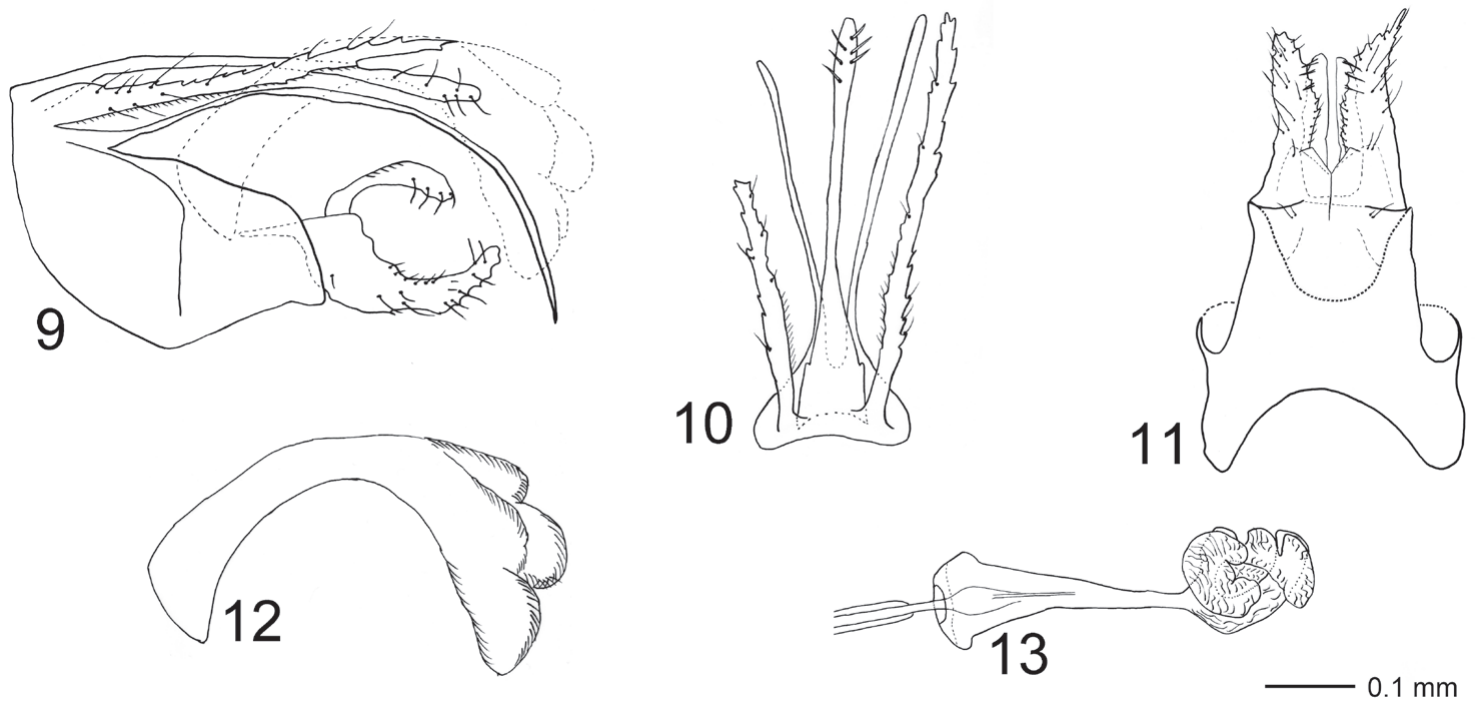
*Triaenodes oscitus* sp. n., holotype. **9** genitalia lateral view **10** genitalia dorsal view **11** genitalia ventral view **12** phallus lateral view **13** phallus ventral view.

Genitalia (Figs 9–13). Segment IX in lateral view with slightly convex anterior margin, dorsal margin short, continuous with dorsal margin of tergum X; ventral two-thirds strongly produced into posterior lobe; posterior lobe with weakly undulating dorsal margin and nearly straight ventral margin except bending ventrally at apex, posterior margin almost truncate. Large triangular, vertical, posteriorly oriented plate located laterally and at mid-length of segment IX, forming narrow, posteriorly orienting processes. In ventral view anterior margin deeply concave; widest before lateral plates, with slightly concave lateral margins; uniformly narrowing after lateral plates. Posterior margins distinct laterally, mesally apparently fusing with gonopods. Cerci elongate, straight, serrated along their lengths, scattered setose, with row of minute setae along anterior three-quarters of ventral margin; in lateral view oriented almost horizontally, basal one-fifth about two times thicker than distal part; in dorsal view slightly diverging, equally wide along their lengths. Tergum X divided into well-developed upper and lower part. Upper part of tergum X slender, about as long as each cercus; slightly curvilinear ventrally along its length in lateral view; basally wide, slender after one-third its length in dorsal view, apex club-shaped, with short, stout setae. Width of base and apex subequal in lateral view. Lower part of tergum X branching at base into two long, needle-shaped processes, clearly longer than upper part of tergum X, curved ventrally along their lengths, reaching further posteriorly than apex of dorsal part of tergum X; in dorsal view slightly diverging. Gonopods each divided into one-branched coxopodite and basomesal process; about two-fifths as long as each cercus. Coxopodites with scattered setae; each with row of smaller setae along central part of dorsal margin; wide at base in lateral view, stepwise narrowing from about one-third, almost sickle-shaped; apex produced dorsally, nearly tangential with phallus. In ventral view, basal one-quarter of coxopodites separated by longitudinal suture, well-separated from one-quarter, each coxopodite forming dorsoventrally flattened, spoon-shaped plate, with serrated mesal margin; apices of coxopodites diverging. Left coxopodite shorter than right coxopodite, apically blunt. Basomesal processes about two-thirds as long as coxopodites; sickle-shaped in lateral view, each process oriented dorsally at base, curving posterad and slightly broadening towards apex, bearing strong, lateroventrally and slightly anteriorly directed spines. In ventral view processes running parallel along their lengths; each process with slightly narrowing apex, mesal margin almost straight, lateral margins with small indentations. Phallic organ widest at base and endotheca, narrowest immediately before endotheca; in lateral view with nearly triangular phallobase; phallotheca slender, widening distally and strongly curving ventrally at about mid-length. In dorsal view endotheca forming irregular lobes.

#### Remarks.

The right cercus is broken at mid-length in the holotype.

#### Material examined.

Holotype male: Fiji: Sigatoka Province, Sigatoka Sand Dunes National Park, coastal forest, 18°10'S, 177°30'E, 10 m, Malaise trap, 22.ix–8.x.2002, leg. M. Irwin, E. Schlinger & M. Tokoka’a. [FNIC, alcohol]

#### Etymology.

*Oscitus*, from Latin *oscito*, gape or open mouth in English, in reference to the shape of the male gonopods in lateral view.

### Triaenodes forcipatus

Puranen Li & Johanson sp. n.

urn:lsid:zoobank.org:act:FECEF693-62B6-4747-B626-531F63F49B43

http://species-id.net/wiki/Triaenodes_forcipatus

Figs 3 , 14–18

#### Diagnosis.

The species is most similar to *Triaenodes kalydon* (Malicky, 2005) from Sumatra, particularly in the male genitalia which comprise two-branched coxopodites and absence of the upper part of tergum X. *Triaenodes forcipatus* is easily distinguished from *kalydon* by having much smaller basomesal processes, downward curving gonopods and longer cerci.

#### Description, male.

Wings (Fig 3). Forewing 5.2–5.9 mm (N=3), hind wing 4.0–4.6 (N=3). Forewing: stem of M absent; forks I and V present; wing membrane with pale area at apex and basal one-third of posterior margin, two rectangular pale fields at anterior margin, well separated by dark area. Hyaline area present at anastomosis. Hind wing: uniformly gray, without apparent patterns.

**Figures 14–18.**
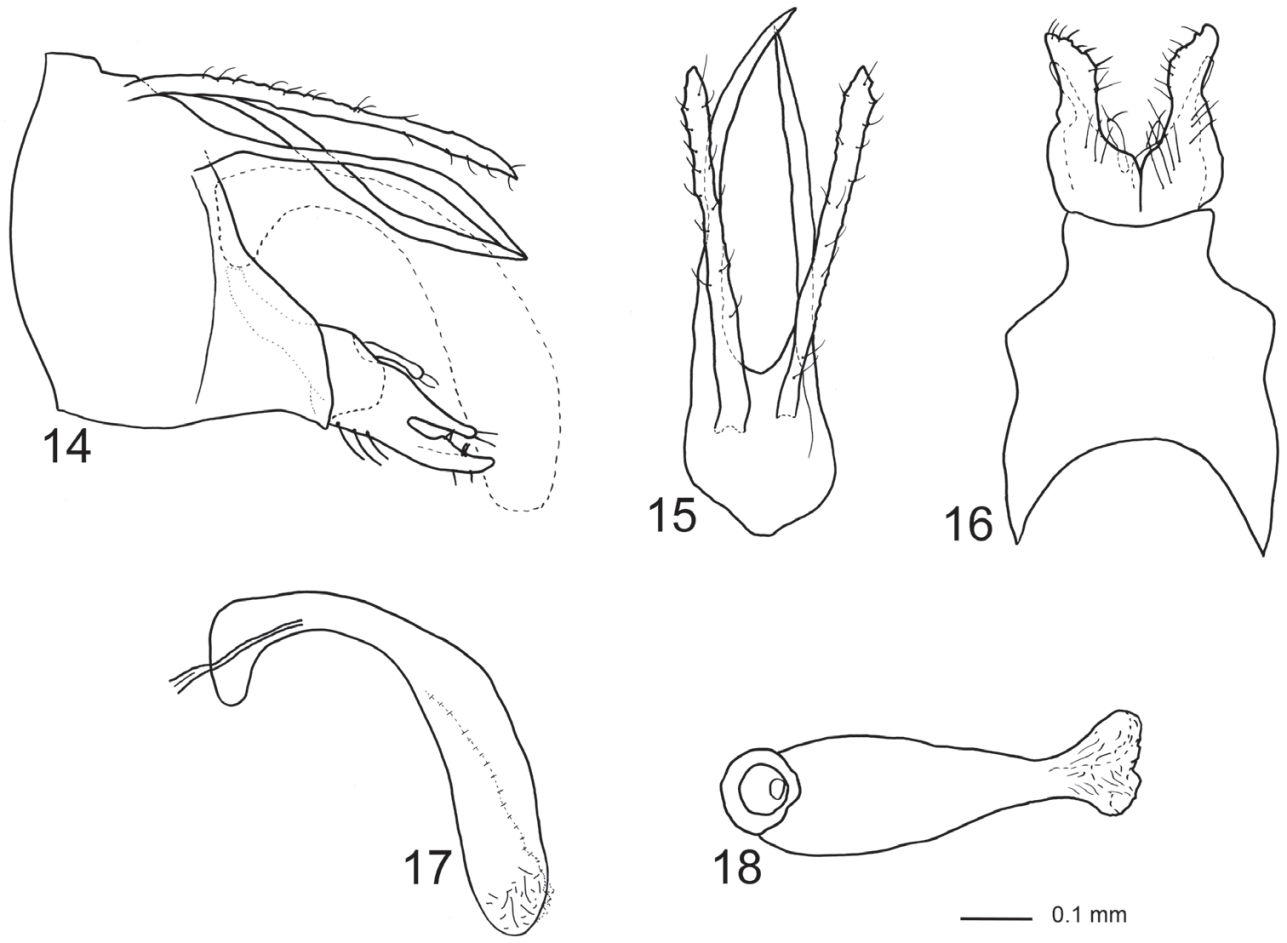
*Triaenodes forcipatus* sp. n., holotype. **14** genitalia lateral view **15** genitalia dorsal view **16** genitalia ventral view **17** phallus lateral view **18** phallus ventral view.

Genitalia (Figs 14–18). Segment IX almost triangular in lateral view, ventrally produced posterad before gonopods; ventral margin two times longer than dorsal margin; in dorsal view slightly longer than wide, central part of anterior margin produced anteriorly into rounded lobe; in ventral view anterior margin deeply concave, posterior margin shallowly concave, anterior two-thirds almost two times wider than posterior one-third, sightly pointed laterad at mid-length. Cerci originating from posterodorsal margin of segment IX, uniformly broad along their lengths, with irregular lateral and mesal margins; setose; apex unevenly narrowing; in lateral view posteriorly slightly exceeding gonopods. Tergum X consisting of lower part, upper part vestigial and not evident; lower part separated from near base into lateral branches slightly longer than cerci, forming a pair of posteriorly elongate processes originating immediately below cerci; basally wide in lateral view, strongly narrowing from basal one-sixth, uniformly slender along posterior five-sixths, apex pointed, proximal two-thirds of left branch nearly straight, distal one-third slightly curving ventrally; right branch sigmoid, with posteriorly orienting distal one-third; connected ventrally by concave plate. Gonopods in lateral view as high as posteriormost part of segment IX, each divided at mid-length into dorsal and ventral branch, dorsal branch about half width of ventral branch, slightly shorter than ventral branch, dorsal branch bearing marginal setae, ventral branch with scattered setae. In ventral view, anterior half of gonopods forming broad plates being completely fused anteriorly, distal half about half as wide as anterior half, slightly curving laterally, two times longer than wide; each with mesal margin sigmoid, apex almost pointed. Basomesal processes as long and wide as ventral branches, originating from anterior one-quarter of gonopods; apex club-shaped with few setae. Phallic organ strongly curving ventrally; in lateral view phallobase slightly elliptic, phallicata widening immediately after phallobase; endotheca rounded; in ventral view distal end of phallotheca about half as wide as proximal end; endotheca rounded, almost triangular, membranous.

#### Material examined.

Holotype male: Fiji: Viti Levu Island, Pabitra, 17.5833°S, 178.0833°E, 1034 m, Malaise trap, 17–20.xi.2003, leg. Delena Veikovi, Wabu Baseline Survey [FNIC, alcohol].

Paratypes: 5 males: Fiji: Viti Levu Island, Vuda Province, Koroyanitu Natural History Park, Savuione Trail, 17°40'S, 177°33'E, 450 m, Malaise trap, 12–19.x.2002, leg. M. Irwin, E. Schlinger & M. Tokoka’a [NHRS, alcohol].

#### Etymologi.

*Forcipatus*, from Latin *forceps*, claw in English, referring to the claw-shaped gonopods in lateral view.

##### Key to the Fijian species of *Triaenodes*, based on characters on male genitalia

**Table:** 

1	Cerci in dorsal view about three times longer than wide	*Triaenodes dubia* Mosely, 1934
–	Cerci in dorsal view more than four times longer than wide (Figs 4, 9, 14)	2
2	Each coxopodite with one branch (Fig. 9)	3
–	Each coxopodite with two branches (Figs 4, 14)	5
3	Uppermost part of tergum X less than five times longer than wide	*Triaenodes fijianus* Mosely, 1941
–	Uppermost part of tergum X more than six times longer than wide (Figs 9, 10)	4
4	In lateral view, ventral branch of coxopodite about as thick as basomesal process (Fig. 9)	*Triaenodes oscitus* Müller & Johansson, sp. n.
–	In lateral view, ventral branch of coxopodite about two times thicker than basomesal process	*Triaenodes manni* Banks, 1936
5	Basomesal process curving ventrad in lateral view; dorsal and ventral branches of coxopodite curving dorsad in lateral view (Fig. 4)	*Triaenodes rebellus* Eriksson & Johanson, sp. n.
–	Basomesal process nearly straight and almost parallel with dorsal and ventral branches of coxopodite in lateral view (Fig. 14)	*Triaenodes fuscipatus* Puranen Li & Johanson, sp. n.

## Supplementary Material

XML Treatment for Triaenodes rebellus

XML Treatment for Triaenodes oscitus

XML Treatment for Triaenodes forcipatus
